# Single-side polypyrrole coated conductive and flexible polyurethane films

**DOI:** 10.55730/1300-0527.3491

**Published:** 2022-08-10

**Authors:** Melis BİLAL, Esma SEZER, Belkıs USTAMEHMETOĞLU

**Affiliations:** Department of Chemistry, Faculty of Science and Letters, İstanbul Technical University, İstanbul, Turkey

**Keywords:** Flexible, polyurethane, polypyrrole, composite, single side conductive film

## Abstract

In this study, single-side polypyrrole (PPy) coated conductive stretchable polyurethane (PU) / PPy composite films were synthesized by chemical oxidative method in the acetonitrile (ACN)/H_2_O interface as a new alternative to two-side coated PU films which have already reported in the literature. One surface of the PU film was contacted with aqueous Py solution and the other surface with cerium ammonium nitrate (CAN) solution in ACN, so that only one surface of the film was coated with PPy and the resulting composite was named PU/PPy (PUP). This composite was characterized by four-point probe conductivity measurement, Fourier-transform infrared spectroscopy (FTIR), differential scanning calorimetry (DSC), scanning electron microscopy (SEM) measurements, and mechanical testing. The thickness of PPy coating was determined as 16 μm, by using polarized microscope imagines of crosssection of film. T_g_ value of the composite was obtained as −60.38 °C and the highest conductivity of PPy coated side of PU was obtained as 3.5 × 10^−4^ S cm^−1^, while the back side of PU film was still insulator.

## 1. Introduction

PU is one of the multipurpose engineering materials in foam, rigid or flexible form and has many applications such as medical, automotive, furniture, packaging, adhesives and construction [1]. In last two decades, conductive polymers (CPs) have been the focus of lots of researches. Among them, PPy, has been focused on mostly, because of its heterocyclic structure, it has a good electrical conductivity, relative ease to synthesis and oxidative stability compared with other conductive polymers [2]. It can be synthesized in different organic solvents and aqueous media by electrochemical [3] or chemical [4] oxidation of Py. However, PPy is a hard, brittle and limited workable solid that is insoluble in solvents due to strong intermolecular interactions between the chains, therefore, it is possible to overcome this problem by preparation of composites like poly(vinylacetate)/polystyrene/PPy [5], PPy/poly(methylemetacrylate) [6] and PPy/poly(vinylchloride) [7].

On the other hand, plenty of attempts have been made to enhance the performances of stretchable electric materials. The most widely employed strategies in attempting to prepare stretchable electric materials are development of new kinds of elastomers and design of new structures from currently available materials. PU composites [8] and blends [9] are one of the favorite materials due to their improved physical and chemical properties. Conductive composites have an important place in composites and with usage of conductive materials, easy synthesized, good conductive materials with lightweight can be prepared [10]. In the studies on PU composites with different conductive materials such as graphite [11], carbon black [12] multiwalled carbon nanotubes (MWCNTs) [13], ferric compounds [14], graphene [15], it has been reported that, addition of conductive materials as a rigid phase to PU matrix increases the young modulus. PU composites can be prepared also with CPs and among these composites, PU/PPy was reported generally in form of films [12], dispersed particles [16], fibers [17] and foams [18]. In generally, chemical polymerization was used for synthesis by using halogen acceptors [19], ferric compounds, ammonium persulphate [20] and CAN as oxidant [21] as well as by electrochemical polymerization [22]. High specific area of CP composites and combination of excellent electrical, electrochemical and optical properties of CPs and mechanical strength and binding characteristics of polymer-based matrix makes them an excellent choice for lots of applications [23]. Due to elastomeric properties of PU, these materials became stretchable electronics and can also be engineered to be useful in applications in many areas such as high-performance sensors [24], tissue engineering [25], rechargeable batteries [26], supercapacitors [27], corrosion-inhibiting [28], antistatic coatings [29], fuel cells [30], EMI shielding materials [31], biomedical applications [32], wearable textiles [33] desalination [34] and so on. They have been reported that the fabricated composites displayed a good electroactivity and high porosity and specific surface area. The largest difficulties, however, are to control and balance the stretchability, conductivity, and sensitivity of these materials.

In all researches on PU/PPy elastomers, PPy was coated onto both surface of PU films or distributed into PU. Although there is a study about one-side PPy coated polyethylene/PPy composites obtained by using FeCl_3_ and Py vapor, with a conductivity of 10^−3^ S cm^−1^ [35], one-side PPy coated flexible PU film is out of our knowledge.

On the other hand, the use of solid electrolyte in organic batteries and capacitors has gained an importance [36]. PU composite, which is conductive on a single surface, can provide a significant advantage in energy storage technology by acting as both a separator and an electrolyte. Besides, the fact that one of the solvents used during the synthesis of the composite is water provides an important advantage in terms of environment and health [16].

The aim of this study is the fabrication of a stretchable conductive PU/PPy elastomer by in situ polymerization. Py is covering only one surface of the PU film, unlike previous studies that use approximately 20%–30% conductive additive or coat both side of the PU film [37, 38]. Coating one surface of the PU film with PPy in a micron thickness with a single step and an easy method will also reduce the cost compared to coating the bulk or two surfaces of the film.

## 2. Experimental

### 2.1. Chemicals

PU films were purchased from Flokser Co. and the thickness of the films were averagely between 0.3 and 0.4 mm. CAN (548.28 MW; 99% purity) was used as oxidant and purchased from Acros Organics Co. Py (67.09 MW; d = 0.96 g/mL) was purchased from Merck KGaA and used without further purification.

#### 2.1.1. Coating of PPy onto one side of PU film

**Figure 1:**
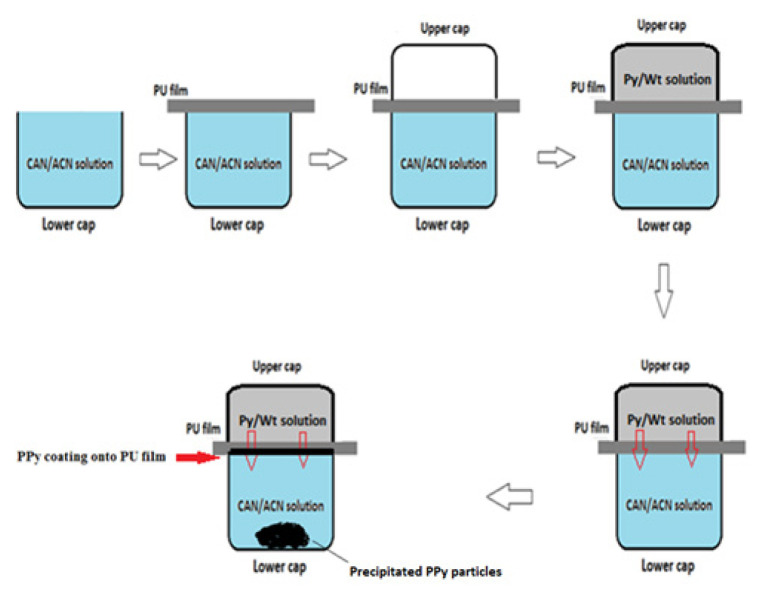
Steps of PPy coating on PU film.

A novel method was used for coating only one side of PU film. First, Py and CAN were dissolved separately in water and ACN, respectively. Then, two solutions were contacted with different surfaces of PU film in vertical direction as shown in Figure 1. Upper and lower cap’s volumes were 6 mL and 24 mL, respectively. The reaction time was 30 min for all experiments. CAN/ACN solution was filled into lower cap and PU film was located over the cap while film and solution in contact. Then the empty upper cap was closed over PU film and Py/water solution was injected into upper cap. During the reaction time, ACN has softened the PU film and Py/water solution diffused to lower cap. Polymerization of Py occurred in lower cap in which, some of PPy coated to PU film’s surface as PPy film while some of them precipitate in solution as PPy powder (Figure 1). Than PUP film was rinsed with water and dried at room temperature.

Py and CAN concentrations, contacted side of solvents, oxidant and solvent types, caps’ position and reaction time were changed to determine the optimum conditions for PUP preparation. The abbreviations of composites according to the effected parameters were summarized in the Table 1.

### 2.2. Measurements

The characteristic functional groups of the PU and PPy and PUP were analyzed with Fourier transform infrared (FTIR) spectroscopy (Perkin Elmer Spectrum One FT-IR Spectrometer, ZnSe crystal model).

The thermal analysis of the PU and PUP were performed by differential scanning calorimetry (DSC; TA instruments, Q10 model) at a heating rate of 10 °C/min and between −80 °C and 150 °C under nitrogen atmosphere. In order to see the effect of the PPy amount on the strength and flexibility of the PUP films, tensile studies were carried out at room temperature using a tensile tester machine, Zwick Roell, BT1-FR 0.5 TH. D14, (Figure S1). The sample was used in a dog-bone type dumb-bell shape. The gauge length and strain rate were 30 mm and 150 mm/min, respectively.

To understand the surface morphology difference between PU, PPy and PUP, scanning electron microscope (SEM, FEI Quanta FEG 200) images were obtained at 50,000 magnification.

Thickness of PPy coatings was determined by crosssection microscope images of PU films and used to calculate the conductivities of PUP. Samples were cut by a cutting device and images were taken by a Leica RM 2265 Fully Automated Rotary Microtome model polarized microscope.

The conductivities of PPy and PUP films were measured by the four-point probe technique using Keithley 2400 model multimeter, Lucas Labs 302 model probe holder and SP4-180-TFS type probe.

## 3. Results and discussion

### 3.1. Steps of PPy coating onto PU film

In the light of previous study [39], Py and CAN concentrations and reaction media were determined. Py was dissolved in water and placed at upper cap as seen in Figure 1, CAN was dissolved in ACN and placed at lower cap. Since the PU film is insoluble in water, first of all, CAN diffuses upwards along the PU film, from the lower solution and Py diffuses downwards from the upper solution forming PPy on the lower surface of the film. This can be achieved only optimum conditions such as, type of oxidant, the concentrations of Py and CAN, effect of caps’ vertical/horizontal positions, solution contact sides of PU film and the reaction time. Conductivities were measured only PPy coated surface of PU films for all these experiments, the other side of films were insulator as expected.

### 3.2. Effect of Py concentration on the conductivity of PU/PPy film

**Figure 2:**
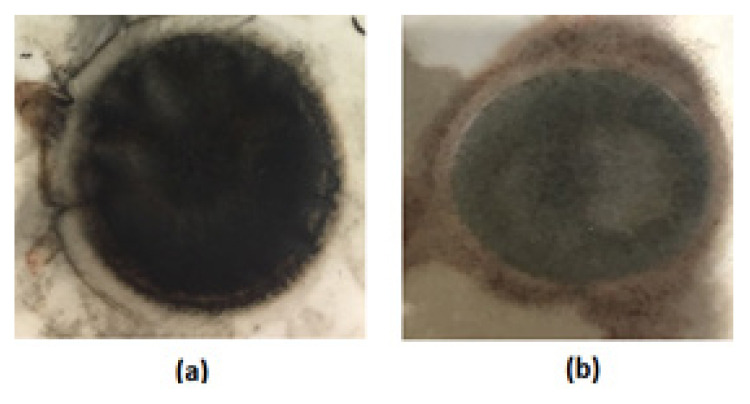
PPy coated lower surface **(a)** and uncoated upper surface **(b)** of the PU film.

PPy can be successfully deposited on the lower part of PU film by adjusting the concentration of Py and CAN. PPy coated lower side and uncoated upper side images of PU film were given in Figures 2a and 2b, respectively. As it can be seen, the PPy coated surface was darker than the uncoated surface as expected.

**Figure 3:**
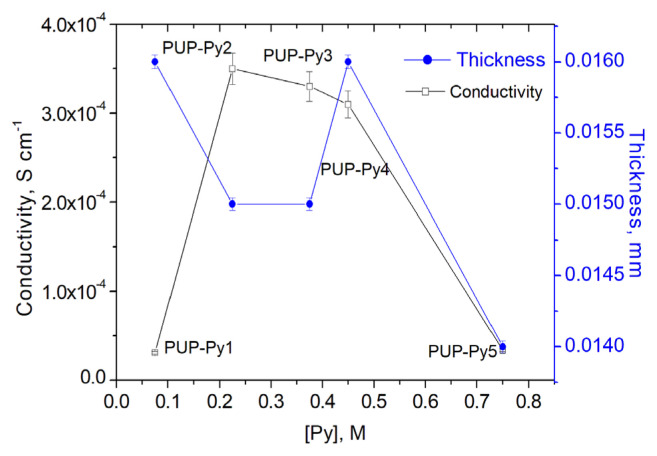
The effect of Py concentration on the conductivities and the thickness of PUP-Py1, PUP-Py2, PUP-Py3, PUP-Py4, and PUP-Py5.

Different Py amounts were studied to investigate the effect of Py concentration on the conductivity of PUP and results were plotted in Figure 3. As it can be seen, the conductivity of PUP increased with increasing Py concentration and reached a maximum conductivity as 3.5 × 10^−4^ S/cm at 0.225 M Py, then started to decrease. After this concentration, some of the PPy began to form in solution rather than on the PU film, as the reaction rate of Py increased kinetically.

The thicknesses of the PPy coating layers on the PU films were measured and the effect of Py concentration on the average thickness of the PPy-coated layer of the PU films was determined using the crosssectional images of the composite films. These thicknesses were used to calculate the conductivity of the composites and plotted versus Py concentration in Figure 3. When the Py concentration was between 0.075 M and 0.375 M, the added Py monomers increased the PPy chain length, increasing the conductivity, while a slight decrease in thickness was observed. When the Py concentration is higher than 0.45 M Py, simultaneous decreases in thickness and conductivity are observed, since PPy formation occurs not only on the surface but also in the solution.

**Figure 4:**
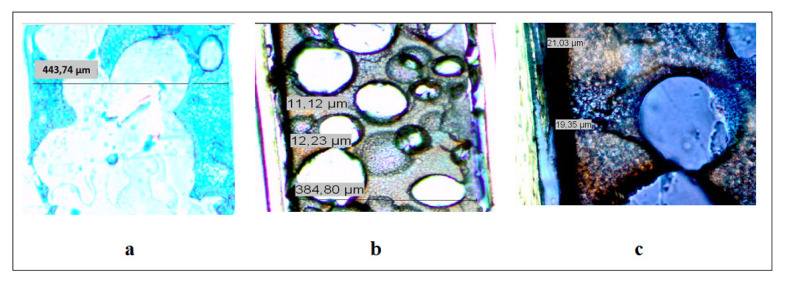
Crosssection microscope images of PU film **(a)**, original view **(b)**, and zoomed view **(c)** of PUP-Py1.

The images of PU, original and zoomed views of PUP-Py1 were given in Figures 4a–4c, respectively, and the thickness of the PU was found as 443.74 μm. The average thicknesses of the PPy layers were found from the original and the zoomed views and obtained as 16 μm. The black line on the left side of the picture indicates the PPy coated conductive side of the film. The thickness of all composite films was calculated similarly.

**Figure 5:**
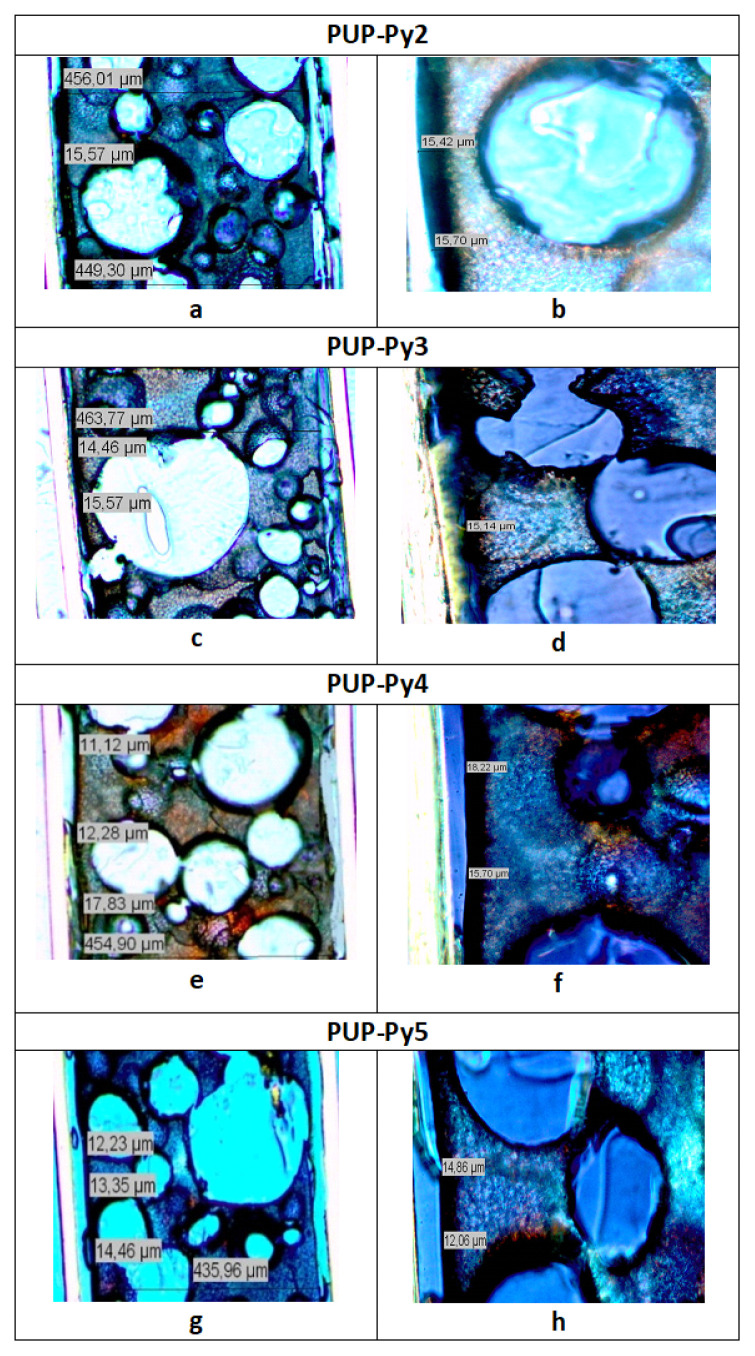
Crosssection microscope images of original **(a, c, e, g)** and zoomed views **(b, d, f, h)** of PUP-Py2, PUP-Py3, PUP-Py4, and PUP-Py5, respectively.

Original and zoomed microscope images of PUP-Py2 which was provided the highest conductivity were given in Figures 5a and 5b. Zoom image of PUP-Py2 looks like the images of PU film. On the other hand, in the images of PUP-Py3 and PUP-Py5, zoom images become darker and observation of the black lines both on the left-hand side and around the circles indicated that PPy coating started to occur through all over the PU film (Figures 5c–5f). This is an undesired situation and might be occurred due to the using of higher concentration of Py and it diffused faster through the PU film.

Although the thicknesses of the of the PPy layers of the films were close to each other, in the case of P PUP-Py5 (Figures 5g and 5h), with increasing of Py concentration, reaction rate increased between Py and CAN and the some of PPy was precipitated in CAN/ACN solution instead of growing onto PU film surface (Figure 1) and conductivity reduced to 3.4 × 10^−5^ S/cm (Figure 3). Conductivity, thickness and color differences of zoomed images suggested that PUP-Py2 is the composited which provided the optimum conditions.

### 3.3. Effect of CAN concentration on the conductivity of PUP film

**Figure 6:**
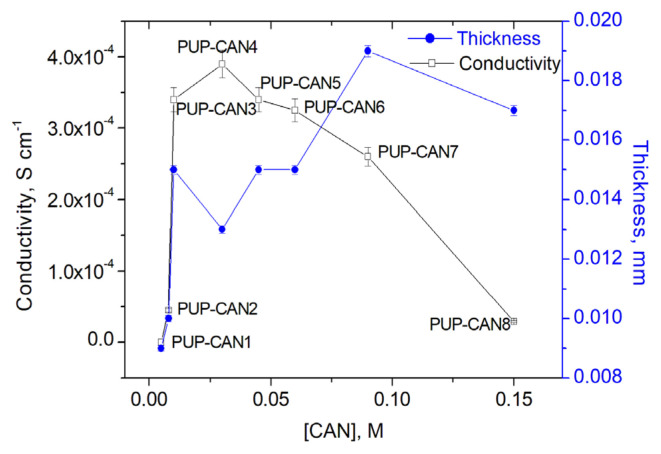
The effect of CAN concentration on the conductivities and the thickness of PUP-CAN1, PUP-CAN2, PUP-CAN3, PUP-CAN4, PUP-CAN5, PUP-CAN6, PUP-CAN7, and PUP-CAN8.

The effects of CAN concentration on the conductivity of PUP were studied and for all experiments, the optimum Py concentration, 0.225 M was used. Experimental procedure was the same with mentioned before (Figure 1). The thicknesses of PU and PPy coatings of the PUP obtained by using different CAN concentration were determined by using the crosssectional images of the composite films. The effect of CAN concentration on the conductivities was represented in Figure 6.

As seen in Figure 6, after having reached a maximum at 0.03 M CAN, conductivity of PUP decreased with increasing CAN concentration. Although there were no significant differences in the thickness of the PPy layers of the films, similar to Py concentration, increase in CAN concentration, resulted some PPy precipitation in solution, instead of deposition on PU film and this caused the decrease in conductivity. It was observed that PPy precipitate was observed in the solution when the CAN concentration was increased as well as when the Py concentration was increased. Obtaining PPy as a precipitate instead of the PPy layer that should be formed on the PU film, as expected, causes the conductivity of the obtained conductive composite to be low. On the other hand, when CAN was used in excess, it is known that some overoxidation and −C=O bonds formation on Py units resulting decrease in conjugated length and conductivity [40]. When the CAN concentration is in the range of from 0.005M to 0.01M, the thickness increases similarly to conductivity, as expected. Up to 0.45M, as the conjugation length of the PPy chain increases, there is some decrease in thickness, as observed under the influence of Py concentration (Figure 3). When the concentration is increased further, the thickness increases as the amount of PPy accumulated on the surface increases, and after 0.09 M CAN concentration, the thickness and conductivity decrease simultaneously because PPy formation begins in the solution.

Although the highest conductivity was obtained in the case of PUP-CAN3 (3.9 × 10^−4^ S/cm), it was observed that the PU film also covered PPy on the back side, with the conductivity of 4.04 × 10^−5^ S/cm which is not desirable (Figure S2). These results showed that the coating of PPy to the only one side of PU needs the suitable mol ratio of Py and CAN. Therefore, PUP-Py2 was selected as optimum PUP.

### 3.4. Effect of different solvents on the conductivity of PUP film

In order to investigate effect of solvents on the conductivity of PU/PPy composite, experiments were repeated with different solvents at optimum condition as in the case of PUP-Py2 and the results are shown in Table S1.

For PU/PPy composite numbered with PUP-S1, PPy precipitated in CAN/water solution not on PU surface, just after transfer of Py/ACN solution to the lower cap. In the case of PUP-S2, PPy coating was not obtained properly since PU film dissolved in THF. For PUP-S3, CAN did not dissolve in THF and PPy coating was observed only on the lower surface of the film. In the case of PUP-S4, PU film which was contacted with CAN/ACN solution at both side, and conductivity was measured as 4.2 × 10^−4^ S cm^−1^. But PUP-S4 was not selected as optimum PUP, because of PPy diffusion into PU film as shown in Figure S3. In the case of PUP-S5, PPy was coated onto PU film in negligible amounts and conductivity could not be measured. According to these results, further investigation has been carried out with PUP-Py2.

### 3.5. Effect of different oxidant on the conductivity of PU/PPy composite film

In order to investigate the effect oxidant on conductivity of PU/PPy composite, another experiment at optimum condition similar to PUP-Py2 was repeated with FeCI_3_ as oxidant and resulting composite was called as PUP-Fe. The conductivity of PUP-Fe was obtained as 3.1 × 10^−4^ S cm^−1^ and was slightly smaller than PUP-Py2. The thicknesses of PPy coatings were determined by crosssection microscope image of PU film (Figure S4) and summarized in Table 2.

Since the color of the image was darker than the color of the image of PUP-Py2 (Figure 5), it seems, when FeCl_3_ was used as oxidant, PPy accumulated on the other surface of film as an unfavorable situation. This may be because the oxidation potential of Fe, which is lower than CAN, allows pyrrole to form on the surface of the PU film rather than form in solution, due to slower polymerization. However, PPy coating on the other side was not enough to measure conductivity and this side still showed insulating property.

### 3.6. Effect of solution contact sides on the conductivity of PUP

In order to see the effect of aqueous and organic solutions used in on the top and bottom of the reaction vessel on the PPy coating, experiments were carried out by changing their places. For this purpose, Py/water solution was placed in the lower chamber and CAN/ACN solution was placed in the upper chamber instead of being used as it was until now. All other parameters were the same with the case of PUP-Py2 and resulting composite was called as PUP-UL. The thicknesses of PPy which was obtained by microscope image of PUP-Py2 and PUP-UL (Figure S5) were 15 μm and 17 μm, respectively. The corresponding PU film thickness of these composites were 450 μm and 440 μm.

PPy coating was occurred again on the surface of PU film which was contacted with Py/water solution instead of CAN/ACN contacted surface. Compared with PUP-Py2, conductivity reduced from 3.5 × 10^−4^ to 2.9 × 10^−5^ S/cm for PUP-UL. This may be because the high-density water phase in the upper chamber also contributes to the reaction under the influence of gravity. Thin PPy coating was formed on the upper surface of the PUP-UL in contact with the CAN/ACN solution. This may be due to the film absorbing a small amount of Py/water solution from the bottom up. However, the amount of coating is not sufficient to measure the conductivity on these surfaces.

### 3.7. Effect of reaction time on the conductivity of PUP film

The effect of reaction time on the formation of PUP was studied as seen in Table 3.

In the case of PUP-T1, conductivity of the composite was 3.2 × 10^−4^ S cm^−1^ which was close to value of PUP-Py2 and the conductivity of PUP-T2 was 4.5 × 10^−4^ S cm^−1^ that is the obtained highest conductivity. However, as shown in Figure S6, Py diffused to the other side of PU film, the coating thickness values of PPy layer were not homogenous and changed from 9 μm to 17 μm. This might be due to the swollen of PU film with CAN solution at longer time of PUP-T2 than PUP-T1 and PUP-Py2, resulted irregular thickness formation. Therefore, further characterizations were continued with PUP-Py2.

Although it was not possible to make one-to-one comparison, because the composite in this manuscript was one-side coated PU/PPy film, and reports on conductive composite preparation by the method used in this manuscript are out of our knowledge, some comparison of the properties with related literature, summarized in Table S2 that was included in the supporting information file. When the conductivity of the one-side coated composite obtained in this study was compared with the conductivity of the composites, it was seen that the conductivity was in a similar range with the conductivity of the electrochemically obtained composites [41, 42]. The reason why the conductivities are similar may be due to the coating on one-side of both composites. On the other hand, when in situ polymerization of Py in PU solution by APS or by nanoblend formation [20], on PU substrates by ferric nitrate and 2-sulfosalicylic acid hydrate [43], on a porous PU film surface by FeCl_3_ [44], by FeCI_3_ in presence of MWCNT and blending with PU[31] performed PPy occurred in the PU matrix and the conductivities of the composites were higher than our case. On the other hand, although chemical polymerization of Py in the host matrix of PU by FeCl_3_.6H_2_O gives the same conductivity with our case, conductivity can be increased only by addition of montmorillonite [45]. The matrix assisted pulsed laser evaporation deposition of PPy [46] allowed to obtain PPy-based composite layers with conductivities up to 10^−2^ S/cm for PPy loadings at 1:10 weight ratios. The highest conductivity, 7.41 S/cm was achieved by the addition of PPy/Fe_3_O_4_ nanocomposite into PU matrix [12]. As can be seen, although PU/PPy composite films with higher conductivities have been reported in the literature, the method proposed in this study is advantageous because it does not contain additives and is a simple and water-based method, considering the complexity of the methods used and the need for additional chemicals.

### 3.8. FTIR results

**Figure 7:**
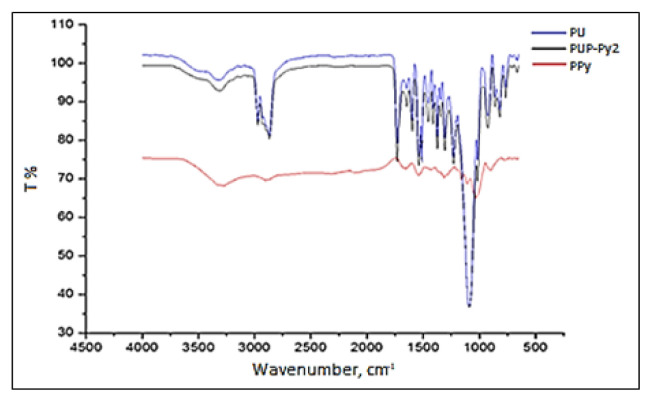
FTIR spectra of the pure PU, PPy and PUP-Py2 composite film.

Figure 7 shows the FTIR spectra of the PPy, PU, and PUP-Py2 films. The characteristic peaks of PU [25], such as the peak at 3305 cm^−1^ due to the −NH stretching vibrations, strong absorption band in the region of 2860–2940 cm^−1^ due to −C–H stretching, the band around 1725 cm^−1^ due to nonbonded −C=O stretching, the band at 1227 cm^−1^ due to −C-O absorption and the band at 1598 cm^−1^ due to −C=C absorption from the aromatic ring [47] appeared in the spectrum of the composite. Also, both of PU and PUP spectra have a sharp peak in 1096 cm^−1^ and belong to COC symmetric stretching vibrations of PU [48].

These peaks are an evidence for PUP formation. On the other hand, PPy spectra have a band at 3300 cm^−1^ due to −NH, the band around 1640–1685 cm^−1^ due to −C-N bond, the band at 1310 cm^−1^ due to the −CH in-plane deformation. The peaks about 1539 cm^−1^ and 1456 cm^−1^ were due to the C=C and C-N stretching, respectively [49]. The peak at 1373 cm^−1^ was due to a NO_3_^−^ ligand incorporated into the polymer by CAN. The increase in the peak intensity at 1310 cm^−1^, by addition of PPy to PU supported successful incorporation of PPy into the PU matrix.

### 3.9. SEM results

**Figure 8:**
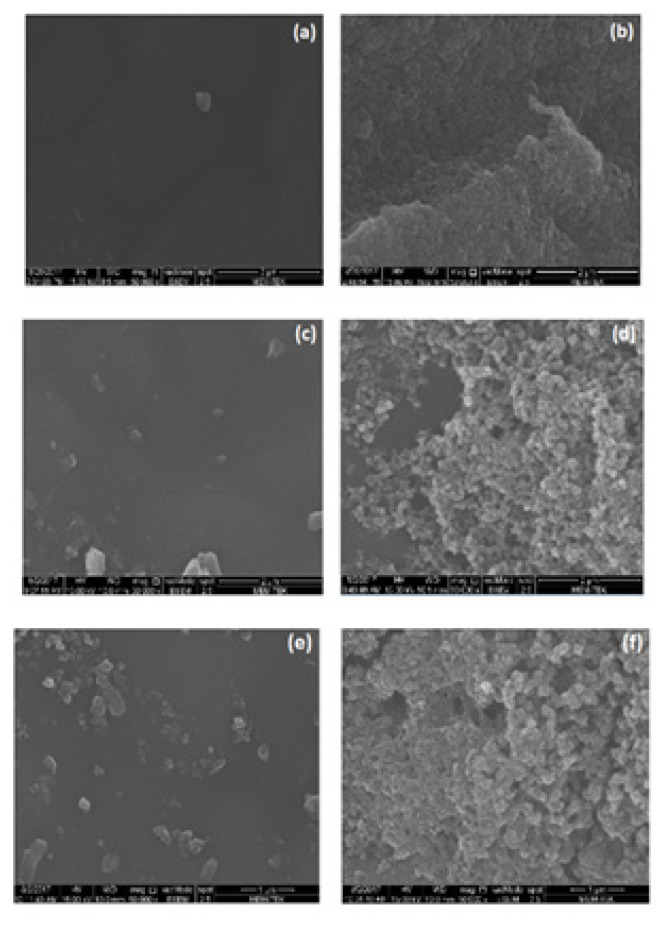
SEM micrographs of PU film **(a)**, PPy **(b)**, uncoated side **(c, e)** and coated surfaces (d, f) of PUP-Py2 and PUP-CAN4, respectively. Magnification: 50,000.

To understand the surface morphology difference between PU, PPy and PUP, SEM images were obtained as shown in Figure 8. As it can be seen from these images, PU exhibited a very smooth surface with few amounts of unreacted white particles (Figure 8a), when PPy was coated onto PU film, well-known cauliflower like morphology of PPy (Figure 8b). Uncoated surface of PUP-Py2 has also a smooth surface like PU (Figure 8c). There were some particles on the surface that could be unreacted particles in PU film or although the conductivity could not measure on that surface that could be PPy which was diffused to other side of PU film. Cauliflower like morphology of PPy was also observed for PUP-Py2 film (Figure 8d). Uncoated surfaces of PUP-Py2 have a smaller number of particles on its surface than PUP-CAN4 as it can be clearly seen in Figures 8c and 8e, respectively. Because of this reason, PUP-CAN4 showed some conductivity at the uncoated side of the film (Figure 6).

### 3.10. DSC results

Thermal behaviors of PU, PUP-Py2 and PUP-Py4 were investigated and were given in Figure S7. PPy shows has no glass transition temperature (T_g_) and the melting temperatures (T_m_), which is a characteristic property of the PPy [46]. The T_g_ value of PU film’s changes from −70 to −25 depending on their hard/soft segments and linkage types [21,50,51], and in this study, it was found as −62.68 °C which is compatible with the literature. T_g_ of PUP-Py2 and PUP-CAN4 were shifted to −60.38 °C and −59.80 °C, respectively, due to PPy coating onto films. An increase in T_g_ of the softer PU phase was observed as a result of the addition of a hard PPy phase to PU as expected.

The Tm values of PU, PUP-Py2 and PUP-CAN4 were detected as 58.69 °C, 62.76 °C and 68.99 °C for PU, PUP-Py2 and C4, respectively. Presence of more amount of rigid PPy phase in the case of PUP-CAN4 than PUP-Py2, resulted in higher T_g_ and T_m_ values than PUP-Py2 as expected. Increases in Tm values of composites as compared to PU can be an indication of the PPy inclusion to PU structure.

### 3.11. Tensile test results

**Figure 9:**
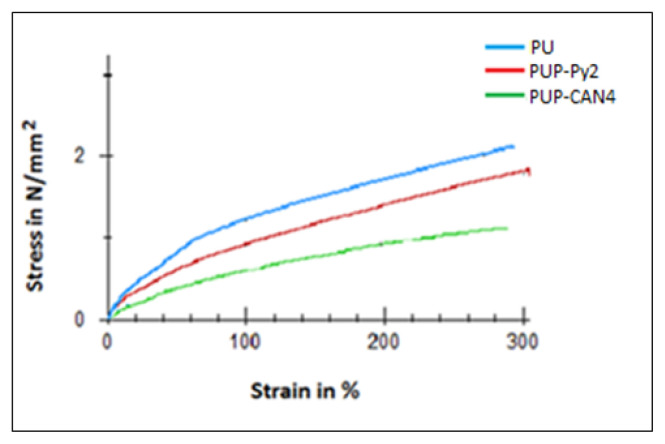
Stress-strain curves of PU, PUP-Py2 and PUP-CAN4.

Mechanical behaviors of PU, PUP-Py2 and PUP-CAN4 were studied. Stress-strain curves and modules and elongation breaks was given in Figure 9 and the results were collected in Table 4. When rigid phase was added to the matrix phase, modulus is expected to increase [17]. Similarly, with addition of a hard PPy phase to a soft PU phase, it was expected to find an increasing value of E-mod than the value PU. However, as seen in Table 4, there was an opposite case in E-mod values and PU has the highest value. This indicates that PU can stretch less than PUP-Py2 and PUP-CAN4 at same stress application to the composite. This counterintuitive result of modulus value can be an explained by softening PU film by CAN during PPy synthesis in the case of PUP-Py2 and PUP-CAN4. Similar unexpected result was reported for PET filaments which were treated with xylene solvent for 20 min [52]. Xylene affected the amorphous regions in polymer chains and a reduced modulus was observed in the case of treated PET.

When comparing the composite (PUP-Py2) coated with PPy on one-side and the composite (PUP-CAN4) coated on both sides, flexibility decreases when two surfaces are coated, as expected.

The E-mod of the composite decreased by 33.63% and 44.84% in the case of PUP-Py2 and PUP-CAN4, respectively. These results were similar to the result obtained for PPy/PU composite in the literature [12].

In order to determine the changes in morphology and the conductivity of PUP under different elongations the conductivities of the PUP films (PUP-Py2 and PUP-CAN4) were measured before and after stretching and the ratios of the difference in conductivity (ΔX) after and before stretching to the one before stretching (X0) were calculated. Although there is no obvious mechanical damage for the PUP when stretched to 300% of its original length, the conductivities decreased with stretching. Moreover, ΔX/X0 depended on the elongation levels. In the case of PUP-Py2, at the 10% elongation, the value was 0.8. The further increase in the elongations to 25% and 100% caused to increase up to 0.9 and 30, respectively. These results are well agreed with the literature findings. It has been reported that the amount of conducting polymers on the surface and the elongation play an important role on the electrical conductivity of conducting elastomers and when they were stretched the conductivity decreases with increase in the strain due to cracks on the surface [43, 53]. The reason of the decreasing conductivity might be the formation of some cracks as suggested in the literature.

## 4. Conclusion

A new type of PU/PPy composite (PUP) has been developed by oxidative polymerization of Py on the surface of PU film. PPy was coated onto PU film by the reaction of aqueous solution of Py contacted to one side of the film with CAN in ACN contacted to the other side. The effects of different Py and CAN concentrations, solvent contact sides, caps’ horizontal/vertical positions were investigated and the optimum condition was determined. PUP films were characterized by four-probe electrical conductivity, optical microscope, FTIR, DSC, SEM and tensile test measurements.

FTIR measurements of PUP showed characteristic peaks of PU and PPy with an increase at peak intensities. The presence of the characteristic peaks of PU and PPy in FT-IR spectra of PUP supported the formation of composite.

Observation of well-known cauliflower like morphology of PPy in the SEM images of conductive side of PUP-Py2 film, increase in T_g_ values for the PUP-Py2 film comparing with PU and decrease of young modulus of PU films after PPy coating were also supported the composite formation.

The thicknesses of PPy coatings were determined by using polarized microscope imagines of crosssection of composites and PPy was coated as 15 μm on the one side of 450 μm PU film and the highest conductivity was achieved as 3.5 × 10^−4^ S/cm while the other surface is still insulator.

In conclusion, with the advantages of good mechanical properties of PU and the conductivity property of PPy, it is possible to obtain the flexible, cost effective, one-side conductive and other side insulator PU/PPy composites which makes it favorable for energy storage technology by acting as both a separator and an electrolyte and it is also a suitable candidate to be used as a new material in flexible electronics such as sensors, smart textiles, wearable device and also flexible solar panels due to their easy fabrication and enhanced mechanical properties.

## Supporting Informations

Figure S1 Tensile test machine with sample at initial point **(a)** and during elongation **(b)**.

Figure S2 Crosssection microscope images of original **(a, c, e, g)** and zoomed views **(b, d, f, h)** of C1, C2, C3, C4, C5 and C6, respectively.

Figure S3 Crosssection microscope images of the S4.

Figure S4 Crosssection microscope image of F.

Figure S5 Crosssection microscope images as original **(a)** and zoomed view **(b)** of D.

Figure S6 Cross-section microscope image of T2.

Figure S7 DSC thermograms of pure PU, PU/PPy composites, P2 and C4.

**Table S1:** Effect of different solvents on conductivity of PU/PPy composite films.

PU/PPy composite no.	Solvent		Contact side to PU film		Conductivity, S cm ^−1^
	Py	CAN	Py	CAN	
P2	Water	ACN	Upper	Lower	3.5 × 10 ^−4^
S1	ACN	Water	Upper	Lower	-
S2	THF	Water	Upper	Lower	-
S3	Water	THF	Upper	Lower	-
S4	ACN	ACN	Upper	Lower	4.2 × 10 ^−4^
S5	ACN	ACN	Lower	Upper	-

**Table S2:** Comparison of conductivities of different PU/PPy composites.

Composite	Conditions	Conductivity (S/cm)	One or two side	Bulk	Ref.
ITO/PU/PPy	Electrochemically	1.0	One	**-**	41
PU/PPy nanofiber	Electrochemically	10 ^−4^–1	One	**-**	42
PU/PPy	PPy/Fe _3_O _4_ NCs was added into the host PU matrix.	7.41	Two	Bulk	12
	In situ polymerization of Py in PU solution	0.07	Two	Bulk	20
	Nanoblend formation	0.26			
	The surface in situ polymerization of Py on PU substrates by Fe (NO _3_) _3_ and sulfosalicylic acid hydrate.	0.036–0.13	Two	Bulk	43
	In situ polymerization of Py with FeCl _3_ in PU film	4	Two	-	44
PU/PPy/MWCNT	In situ polymerization of Py by FeCI _3_ in presence of MWCNT and blending with PU	10 ^−3^	Two	Bulk	31
PU and Montmorillonite/PPy	Chemical polymerization by FeCl _3_.6H _2_O in the presence of montmorillonite	3.79 10 ^−1^	Two	Bulk	45
	Chemical polymerization by FeCl _3_.6H _2_O in the absence of montmorillonite	2.77 10 ^−4^			
PU/PPy	Matrix assisted pulsed laser evaporation deposition of PPy	10 ^−2^	Two	Bulk	46
	PPy/Fe _3_O _4_ NCs was added into the host PU matrix.	7.41	Two	Bulk	12
PUP-Py2	Chemical polymerization by CAN	3.5 × 10 ^−4^	One	-	This study

[12] Mathad JK, and Rao R. Characterization Studies on PoIypyrrole-Fe3O4-Polyurethane Nanocomposite Conductive Films. Journal of Polymeric Materials 2012; 29: 127–136.

[20] Kotal M, Srivastava S, K. and Paramanik B. Enhancements in Conductivity and Thermal Stabilities of Polypyrrole/Polyurethane Nanoblends. Journal of Physical Chemistry C 2011; 115: 1496–1505.

[31] Gahlouta P, Choudhary V. Preparation of highly conductive polyurethane / polypyrrole composite film for flexible electric heater. Synthetic Metals 2020; 266: 116414.

[41] Wang Y, Sotzing GA, Weiss RA. Preparation of Conductive Polypyrrole-Polyurethane Composite Foams by in-situ Polymerization of Pyrrole. Chemistry of Materials 2008; 20: 2574–2582.

[42] Chiu HT, Lin, JS and Huang LT. The processing and mechanical properties of Polypyrrole-Polyurethane alloy films. Journal of Applied Electrochemistry 1992; 22: 528–534.

[43] Li M, Li H, Zhong W, Zhao Q, Wang D. Stretchable Conductive Polypyrrole/Polyurethane (PPy/PU) Strain Sensor with Netlike Microcracks for Human Breath Detection. Applied Materials and Interfaces 2014; 6: 1313–1319.

[44] Xie J, Pan W, and Guo Z. Preparation of highly conductive Polyurethane-Polypyrrole composite film for flexible electric heater. Journal of Elastomers and Plastics. 2021; 53(2): 97–109.

[45] Vargasa PC, Merlini C, Ramoa SDAS, Arenhart R, Barraa GMO, Soares BG. Conductive Composites Based on Polyurethane and Nanostructured Conductive Filler of Montmorillonite/Polypyrrole for Electromagnetic Shielding Applications. Materials Research. 2018; 21(5). https://doi.org/10.1590/1980-5373-MR-2018-0014.

[46] Pauna IA, Acasandrei AM, Luculescub CR, Mustaciosuc CC, Ionb V, Mihailescua M, Vasilea E, and Dinescub M, MAPLE deposition of polypyrrole-based composite layers for bone regeneration. Applied Surface Science Part A 2015; 357: 975–984.

**Table S3:** The ratios of the difference in conductivity (ΔX) after and before stretching to the one before stretching (X_0_) were calculated of PUP at different strains.

PUP	ΔX/X _0_ at 10%	ΔX/X _0_ at 25%	ΔX/X _0_ at 100 %
**PUP-Py2 (front side)**	0.8	0.9	30
**PUP-CAN4 (front side)**	0	0.3	31
**PUP-CAN4 (back side)**	0	0.15	3

## Figures and Tables

**Table 1:** The abbreviations of composites according to Py, CAN and FeCl_3_ concentrations, solvent type, cup position, solution contact sides and time.

Parameters	Py	CAN	FeCl_3_	Solvent	Cup position	Solution contact sides	Time
**Abbreviations**	PUP-Py#	PUP-CAN#	PUP-Fe#	PUP-S#	PUP-HV#	PUP-UL#	PUP-T#

**Table 2:** Thickness of PU films and PPy coatings of PUP films obtained with different oxidants.

PUP	[Py]	Oxidant		Thickness (μm)	
		[CAN]	[FeCl_3_]	PU film	PPy coating
PUP-Py2	0.225	0.01	-	450	15
PUP-Fe	0.225	-	0.01	480	16

**Table 3:** Thickness of PU films and PPy coatings and conductivities of PUP films obtained with different reaction times.

PUP no	Thickness (mm)		Conductivity, S cm^−1^ ×10^−4^	Time (min)
	PU film	PPy coating		
PUP-T1	0.42	0.015	3.2	15
PUP-Py2	0.45	0.015	3.5	30
PUP-T2	0.46	0.012	4.5	60

**Table 4:** Elastic modulus (E-mod) and elongation (%) at break values of PU, PUP-Py2 and PUP-CAN4.

	E-mod (N/mm^2^)	Elongation at break (%)
**PU**	4.46	290
PUP-Py2	2.96	300
PUP-CAN4	2.46	290
