# Supplementary figures and images for: Single-side polypyrrole coated conductive and flexible polyurethane films
Source: Turk J Chem. 2022 Aug 10;46(6):1918–29. doi: 10.55730/1300-0527.3491 (PMC10446928; doi:10.55730/1300-0527.3491)

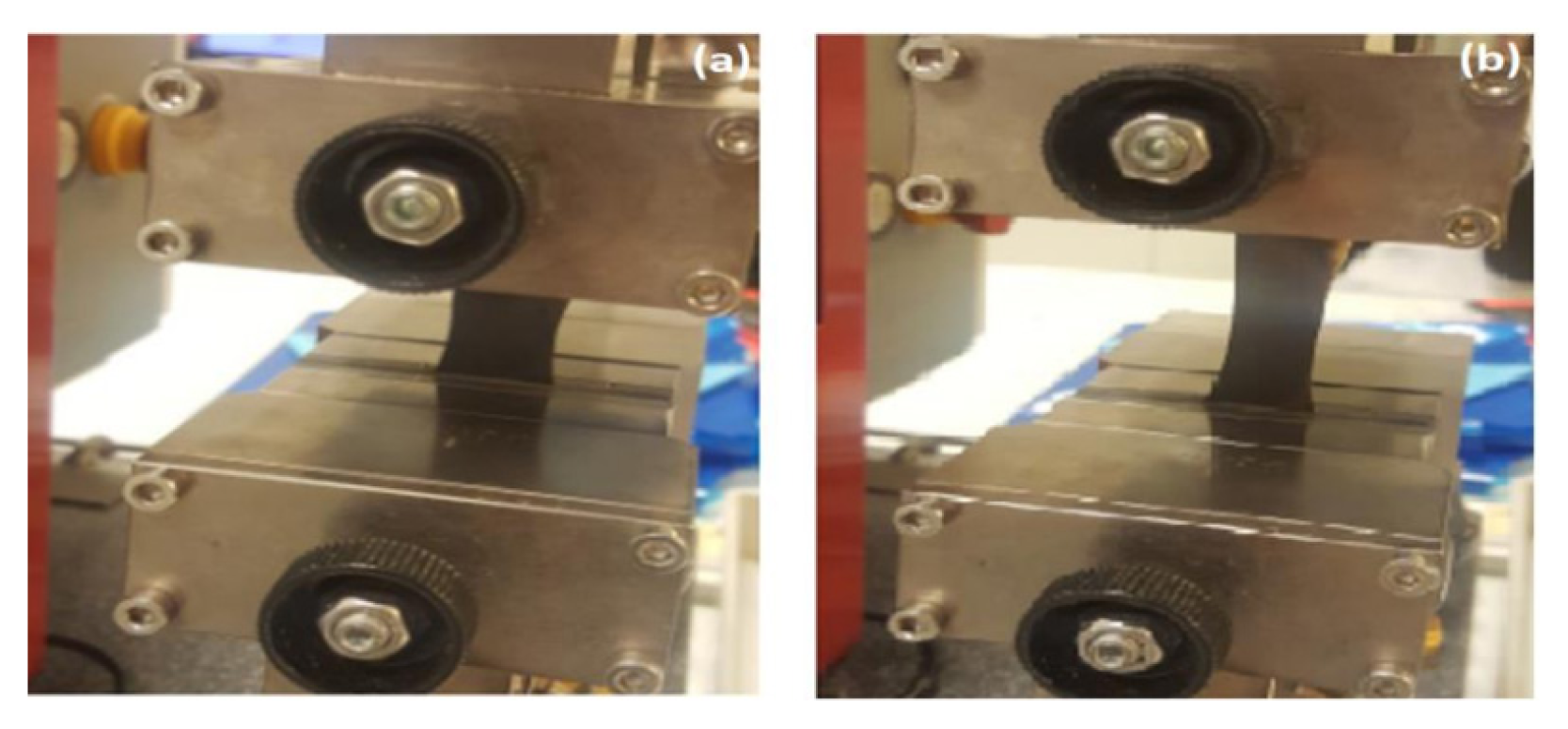

Supplement: Figure S1 — Tensile test machine with sample at initial point (a) and during elongation (b). [file turkjchem-46-6-1918s1.tif]

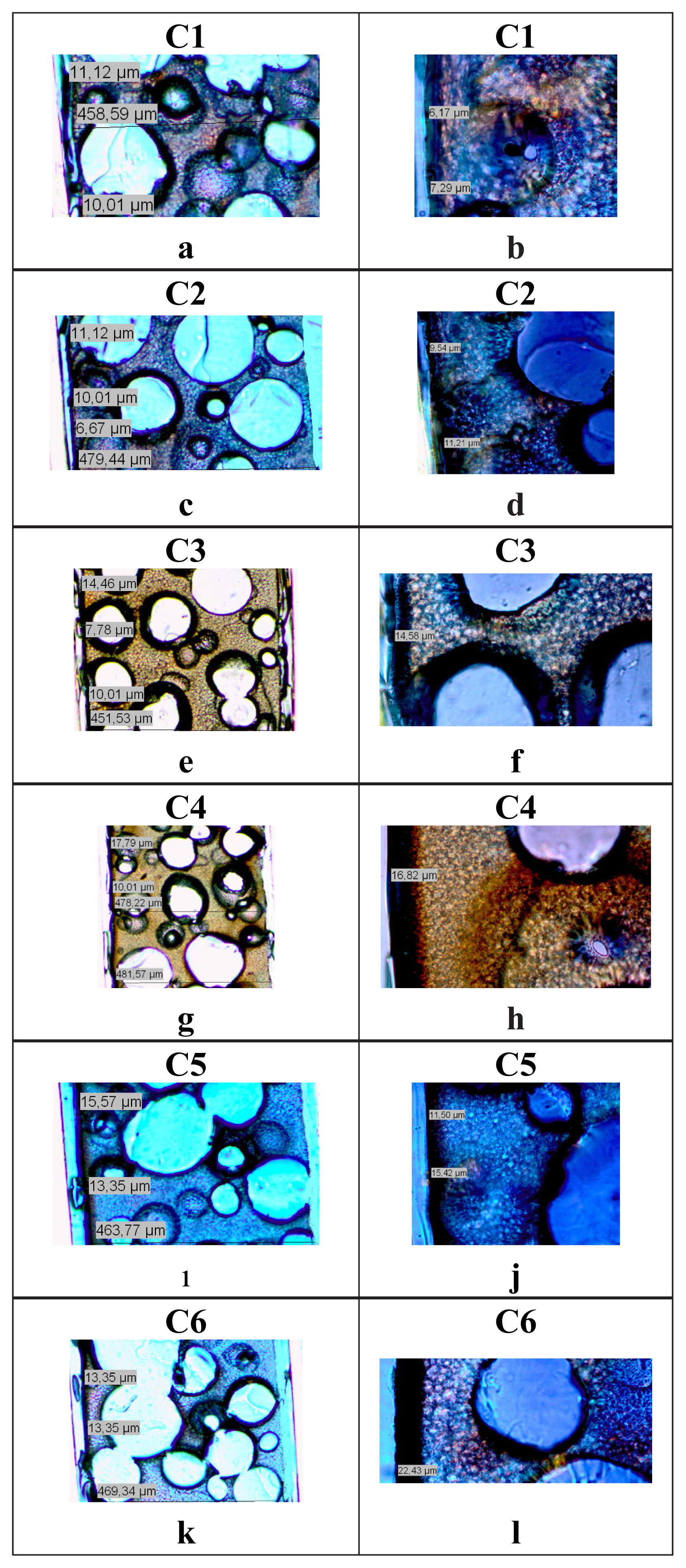

Supplement: Figure S2 — Crosssection microscope images of original (a, c, e, g) and zoomed views (b, d, f, h) of C1, C2, C3, C4, C5 and C6, respectively. [file turkjchem-46-6-1918s2.tif]

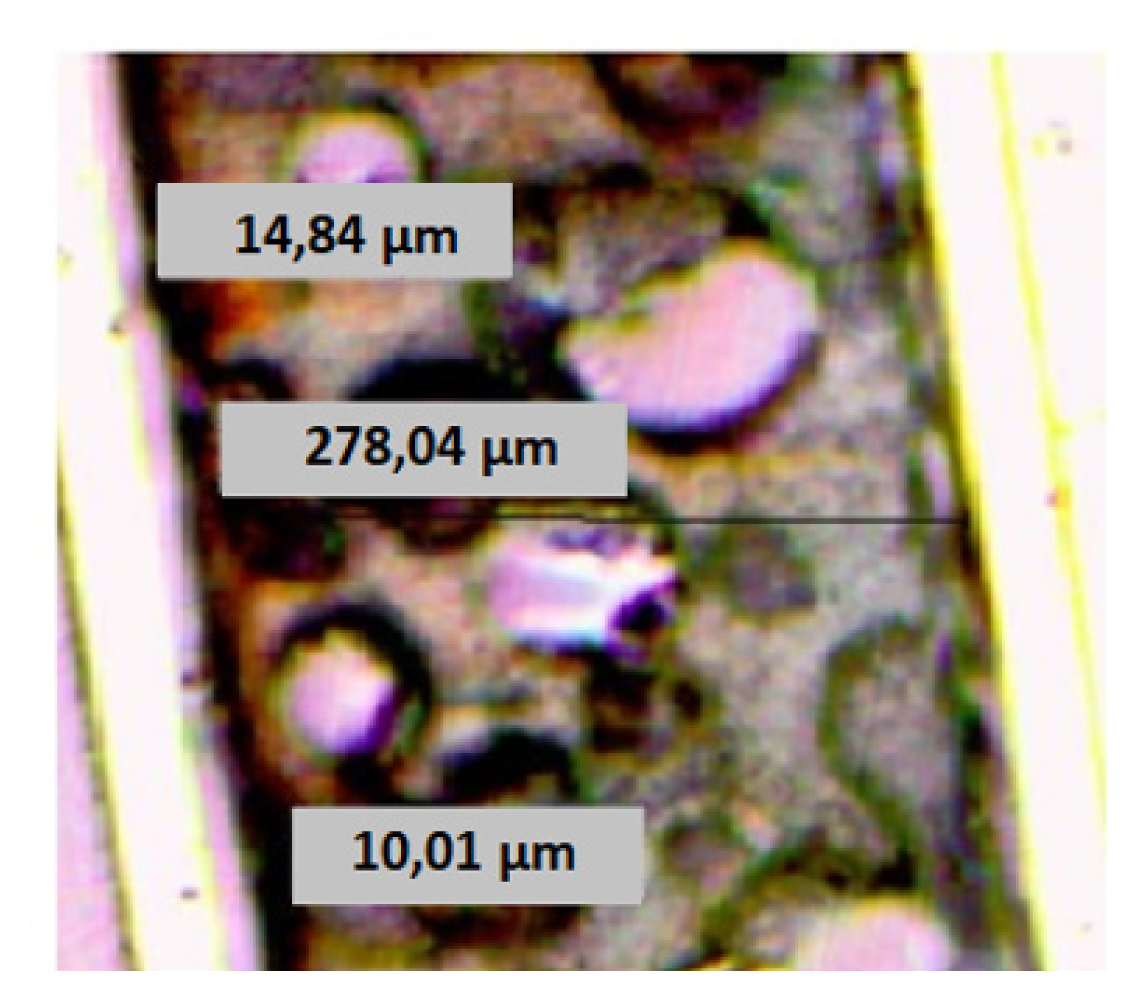

Supplement: Figure S3 — Crosssection microscope images of the S4. [file turkjchem-46-6-1918s3.tif]

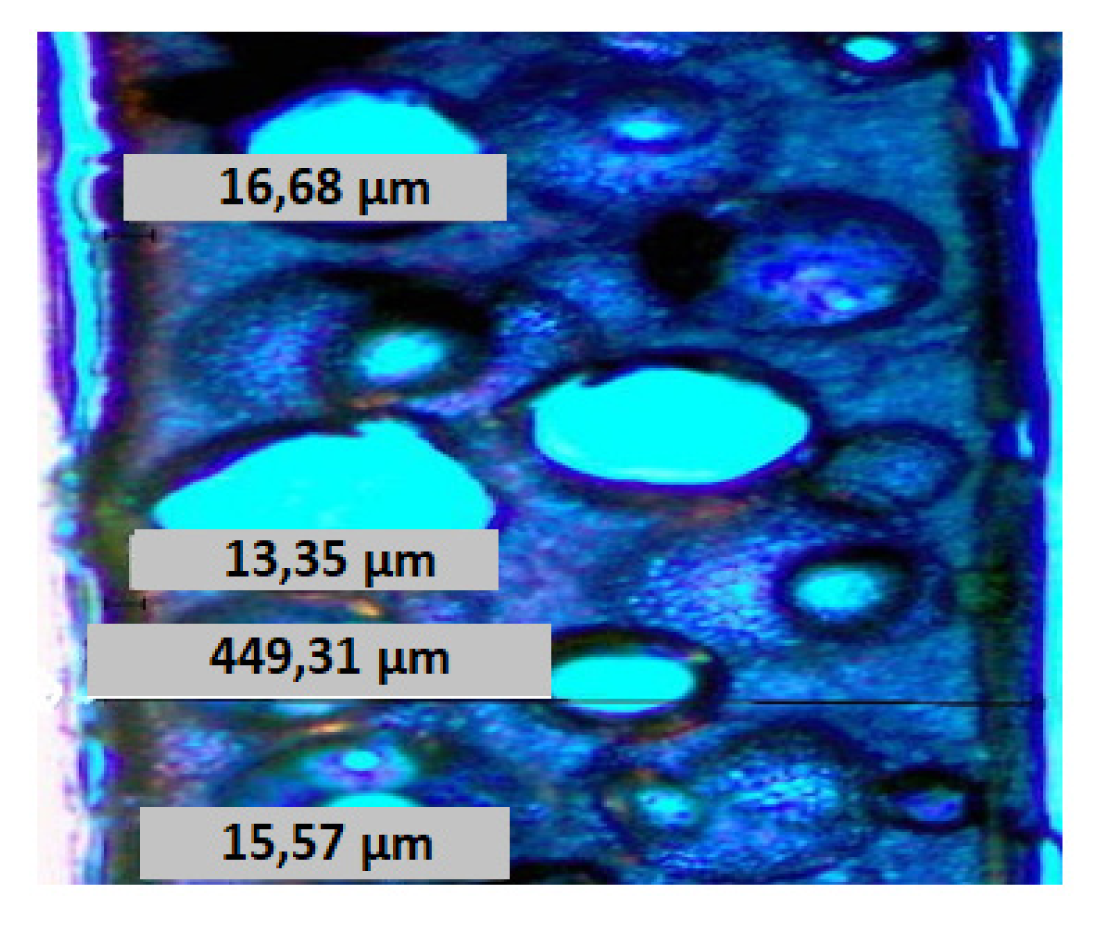

Supplement: Figure S4 — Crosssection microscope image of F. [file turkjchem-46-6-1918s4.tif]

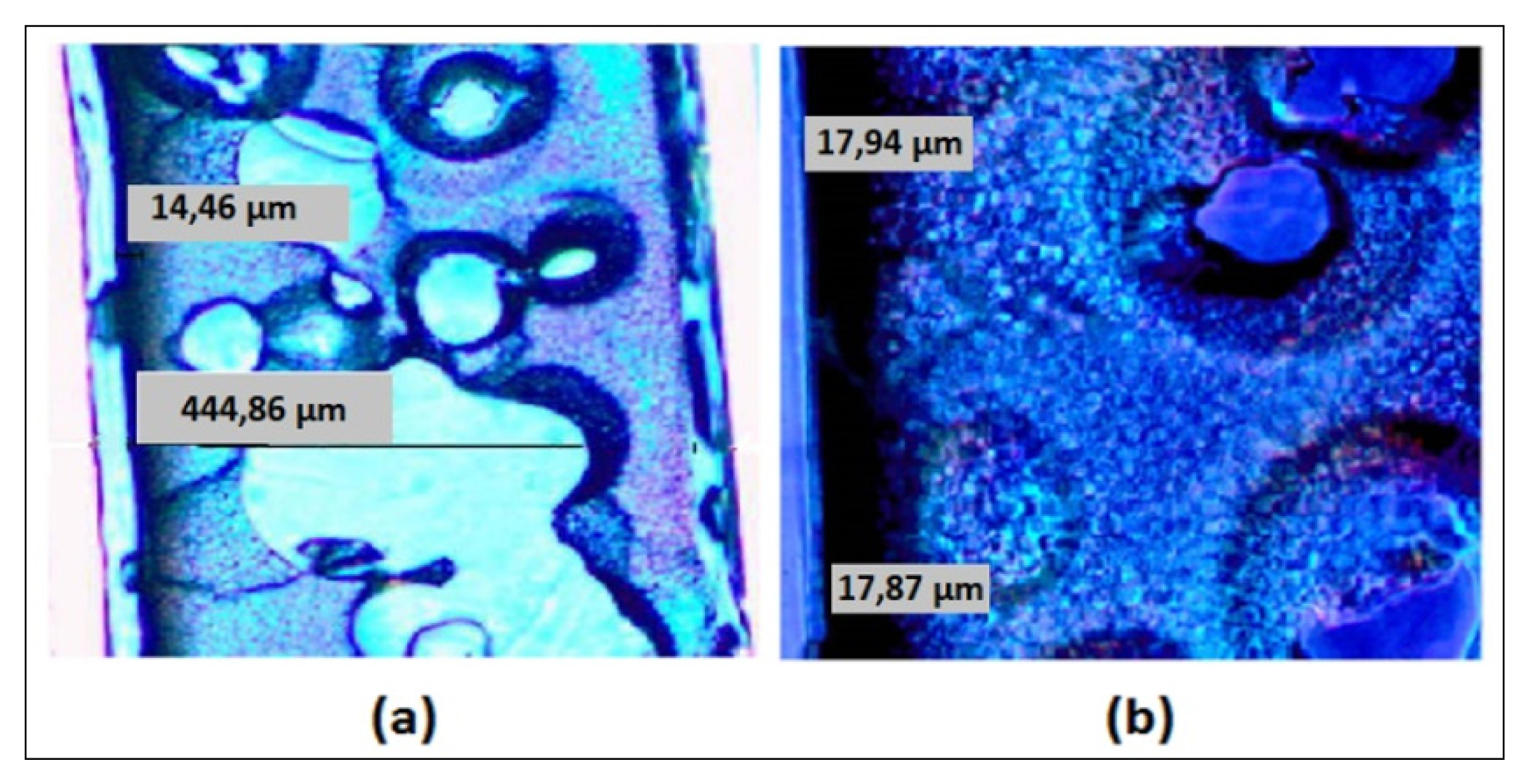

Supplement: Figure S5 — Crosssection microscope images as original (a) and zoomed view (b) of D. [file turkjchem-46-6-1918s5.tif]

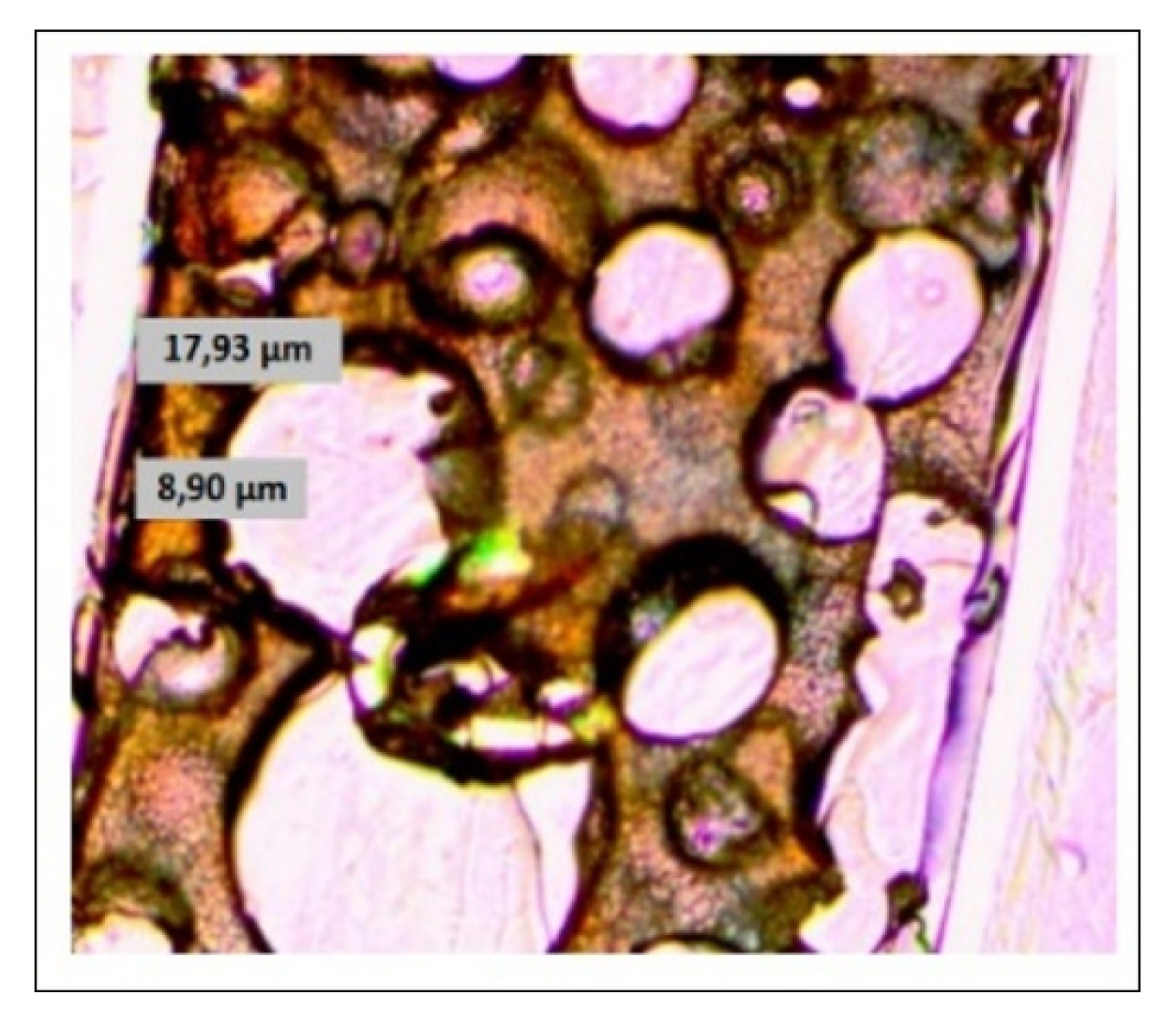

Supplement: Figure S6 — Cross-section microscope image of T2. [file turkjchem-46-6-1918s6.tif]

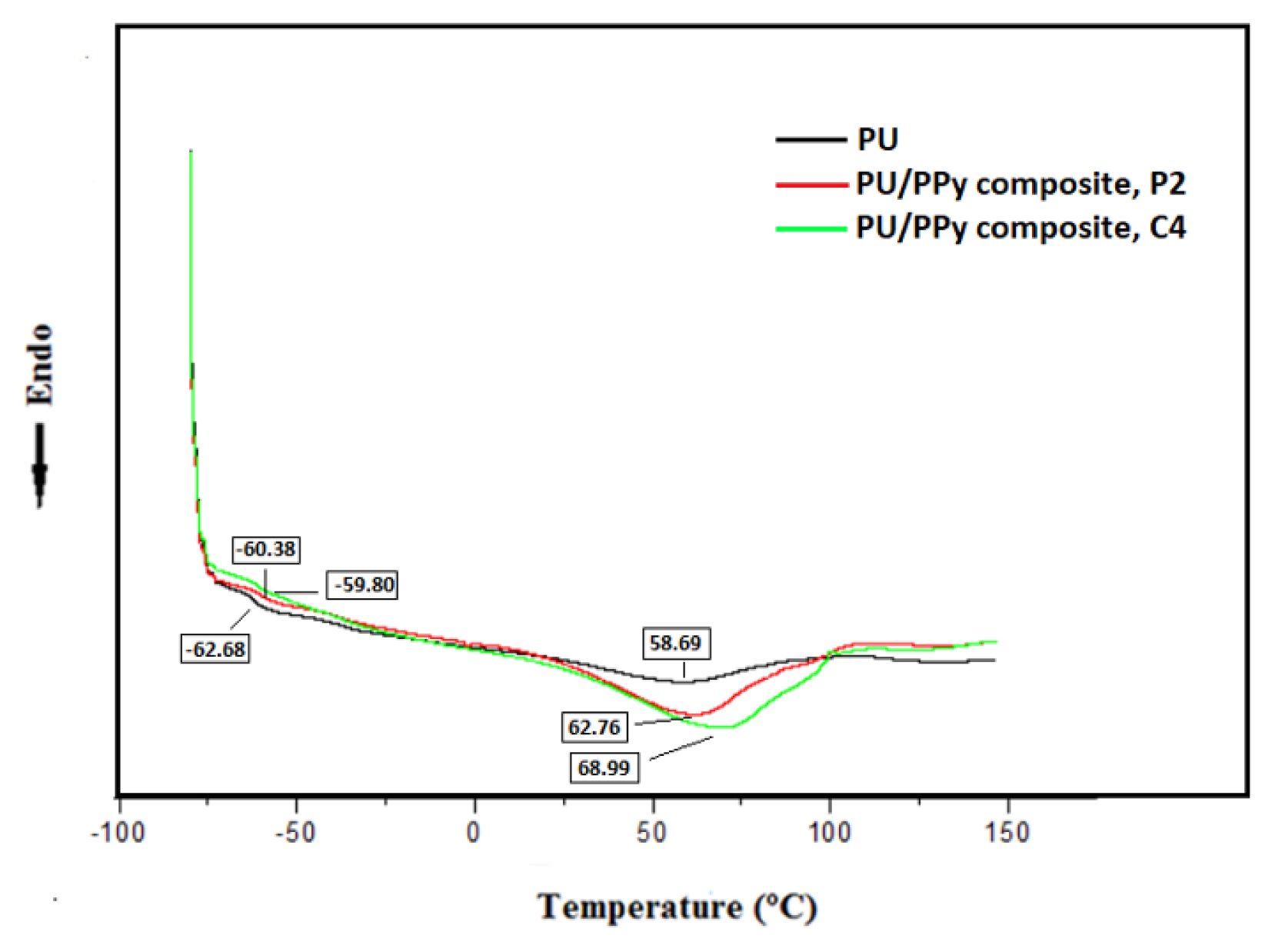

Supplement: Figure S7 — DSC thermograms of pure PU, PU/PPy composites, P2 and C4. [file turkjchem-46-6-1918s7.tif]
